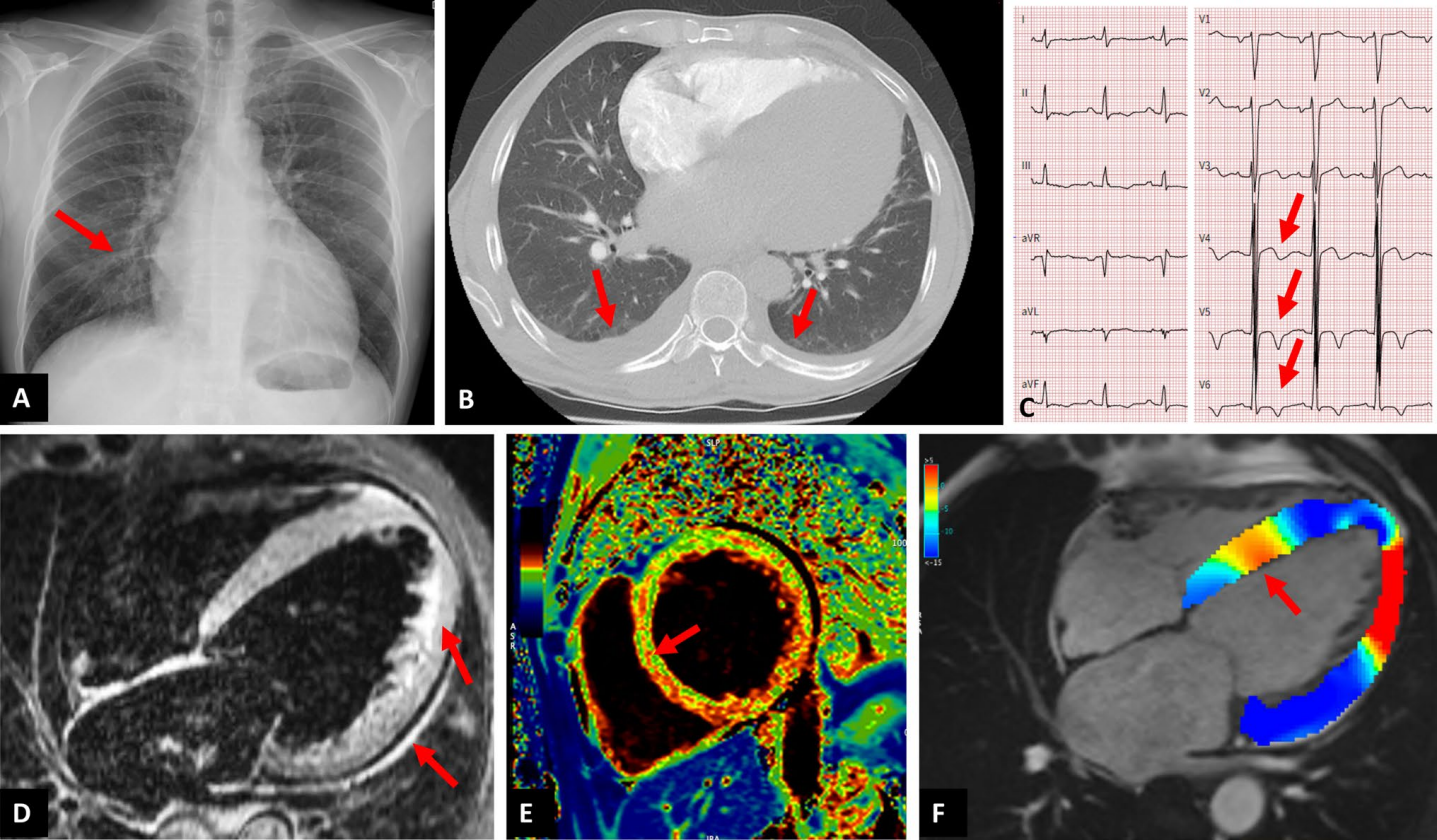# Delayed isolated peri-myocarditis in a Covid-19 patient with respiratory symptoms but without lung involvement

**DOI:** 10.1007/s10554-020-01943-0

**Published:** 2020-07-28

**Authors:** Giancarlo Spano, Kady Fischer, Cédric Maillat, Grégory Vicario, Adrian T. Huber, Christoph Gräni

**Affiliations:** 1 Bern University Hospital, Bern, Switzerland; 2 Hopital du Jura Bernois St. Imier, Saint-Imier, Switzerland; 3 University Hospital Bern, University of Bern, Bern, Switzerland

A 49-year-old prior healthy male without any cardiovascular risk factors living in the western part of Switzerland, noted in mid of March 2020 anosmia and dysgeusia, similar to his wife and four people from his close family, with whom he had frequent contact and who were positive for SARS-CoV-2. Six weeks later, he presented to the hospital with new-onset of dyspnea NYHA 3, general weakness, intermittent epigastrical pain and nocturia without orthopnea nor fever. The retro-nasal SARS-CoV-2 PCR, 6 weeks after initial anosmia and dysgeusia was negative but the antibody IgG blood test for SARS-CoV-2 was positive. Computed tomography of the lungs showed no pulmonary embolism, no infiltrates but left heart congestion, suspected by previous thoracic X-ray (A) and pleural effusion (B). Echocardiography revealed diffuse hypokinesia with severely depressed left- and right-ventricular function. The patient showed elevated C-reactive protein, troponin and NT-proBNP. ECG showed dynamic T-wave changes (C) and after ruling out coronary artery disease, he was diagnosed with isolated peri-myocarditis using multiparametric cardiac magnetic resonance imaging (CMR). CMR showed diffuse thickening of the myocardium and pericardium due to edema confirmed with T2 weighted imaging and T2 mapping (D). Further, pericardial effusion could be seen and tissue characterization revealed diffuse LGE, elevated T1 mapping values and an elevated extracellular volume fraction of 38% (normal value: < 30%), indicating diffuse fibrosis (E). Global myocardial strain of all heart chambers was diffusely impaired (i.e. peak global longitudinal RV-strain of − 11.6% and LV-strain of − 8.7; normal LV-strain value: < − 15%) (F). No other cause was found as an underlying reason for the peri-myocarditis. In the clinical setting of suspected Covid-19 with respiratory symptoms and negative pulmonary imaging, elevated C-reactive protein and troponin should lead to the suspicion of isolated peri-myocarditis. CMR is the primary noninvasive imaging tool to assess peri-myocarditis, also in Covid-19 patients [1, 2].